# Mitochondrial Dysfunction at the Intersection of CKM Syndrome: Molecular Mechanisms and Path-to-Target Therapies

**DOI:** 10.3390/ijms27094120

**Published:** 2026-05-05

**Authors:** Yen-Jung Kuo, Li-Feng Chen, Yumay Chen, Phang-Lang Chen, Hugo Y.-H. Lin

**Affiliations:** 1 General Division, Kaohsiung Medical University Hospital, Kaohsiung 807, Taiwan; q23714073@gmail.com; 2 Department of Medicine, College of Medicine, Kaohsiung Medical University, Kaohsiung 807378, Taiwan; daniel871102@gmail.com; 3 Division of Endocrinology, Department of Medicine, School of Medicine, University of California, Irvine, CA 92697, USA; yumayc@uci.edu; 4 Department of Biological Chemistry, School of Medicine, University of California, Irvine, CA 92697, USA; plchen@uci.edu; 5 Division of Nephrology, Department of Internal Medicine, Kaohsiung Medical University Hospital, Kaohsiung 80756, Taiwan

**Keywords:** mitochondria, CKM syndrome, cardio-renal-metabolic axis

## Abstract

The American Heart Association (AHA) recently formalized cardiovascular–kidney–metabolic (CKM) syndrome to characterize the systemic interplay among cardiovascular failure, chronic kidney disease (CKD), and metabolic disturbances. Despite evolving clinical management, identifying a unifying cellular driver of this multi-organ deterioration remains a critical priority. This review explores the hypothesis that mitochondrial dysfunction serves as the fundamental pathological nexus of CKM syndrome, driving the progression from early-stage metabolic risk to end-stage organ failure. We synthesize evidence demonstrating how nutrient overload and lipotoxicity precipitate a vicious cycle of bioenergetic failure. In the cardiovascular system, ATP deficiency and impaired mitophagy lead to the structural remodeling observed in both heart failure with preserved ejection fraction (HFpEF) and heart failure with reduced ejection fraction (HFrEF). In the kidney, the high mitochondrial density of proximal tubules renders them uniquely susceptible to oxidative stress and mitochondrial DNA (mtDNA) leakage, which subsequently triggers systemic inflammation. Furthermore, we analyze how established therapies—including sodium–glucose co-transporter 2 (SGLT2) inhibitors, Glucagon-like peptide-1 (GLP-1) receptor agonists, and non-steroidal mineralocorticoid receptor antagonists (MRAs)—exert organ-protective effects via mitochondrial mechanisms, promoting metabolic efficiency, reducing reactive oxygen species generation, stabilizing mitochondrial integrity, and promoting mitochondrial quality control processes. Finally, we review emerging mitochondrial-targeted strategies, such as mitoquinol, elamipretide and NAD+ boosters, which aim to restore the SIRT1-PGC-1 α signaling axis. Mitochondria function as the central engines of the CKM axis. A shift toward a mitocentric clinical model may enable earlier intervention and more precise targeting of the mechanisms driving organ crosstalk. Future success depends on multidisciplinary collaboration and the validation of mitochondrial biomarkers to advance precision medicine in CKM syndrome.

## 1. Introduction: The CKM Paradigm Shift

The landscape of chronic disease management is currently undergoing a fundamental transformation. For decades, clinical medicine has operated within isolated specialties, such as treating obesity, type 2 diabetes, CKD, and cardiovascular disease (CVD) as distinct entities with independent pathologies. However, the AHA has recently formalized a more integrative framework: CKM syndrome [1]. This designation recognizes the shared biological pathways and progressive nature linking metabolic dysfunction to end-organ damage.

**Figure 1:**
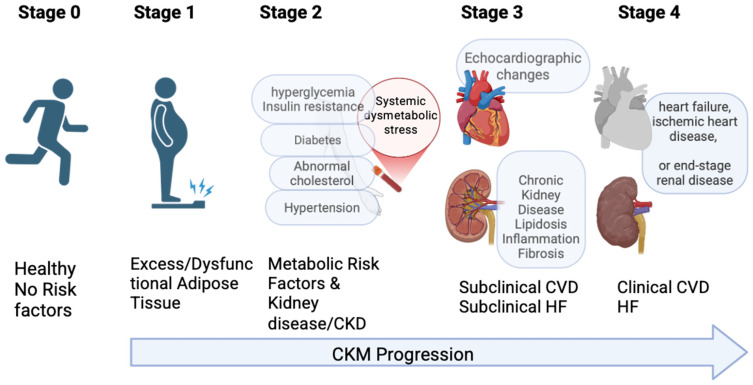
The Cardiovascular–Kidney–Metabolic (CKM) Syndrome Progression Continuum. This figure depicts the multi-stage progression of CKM syndrome, tracing the transition from metabolic health to end-stage organ failure. This trajectory is driven by a systemic dysmetabolic milieu that promotes progressive, interconnected multi-organ dysfunction.

The CKM spectrum follows a predictable, multi-systemic trajectory. It typically begins with stage 1 (overweight/obesity), characterized by dysfunctional adipose tissue, which precipitates systemic insulin resistance [2,3]. This metabolic derangement evolves into stage 2 (diabetes), where hyperglycemia and lipotoxicity begin to exert chronic pressure on the microvasculature [4]. By stage 3, the synergy of hypertension and metabolic stress culminates in CKD [5,6], characterized by a declining glomerular filtration rate and albuminuria. Finally, stage 4 represents the clinical manifestation of overt CVD, including heart failure, coronary artery disease, and stroke [7] (Figure 1).

The clinical significance of moving beyond single disease management cannot be overstated. By treating these conditions in isolation, clinicians often address symptoms too late in the disease progression. The CKM framework advocates for early, holistic intervention, acknowledging that the cardiac health is inextricably tied to the metabolic status and the renal filtration capacity.

However, identifying a clinical framework is only the first step; understanding the underlying biological drivers represents the next challenge. While systemic inflammation and oxidative stress are frequently implicated in CKM syndrome pathophysiology [8,9,10], current evidence suggests they function both as early mediators and as amplifiers within a broader network of cellular dysfunction. In this context, mitochondrial perturbation emerges as a central integrative hub linking metabolic stress, inflammatory signaling, and bioenergetic failure across disease stages. This review therefore proposes that mitochondrial dysfunction represents a unifying pathological axis of CKM syndrome, contributing to both the initiation and progression of multi-organ dysfunction. As the central hub for energy production, calcium signaling, and redox balance, the mitochondrion is both the first responder to metabolic overload and the primary casualty of the resulting systemic stress. By positioning mitochondrial dysfunction at the core mechanism of CKM syndrome, we can identify novel therapeutic targets that offer protection across the entire cardio-renal-metabolic axis.

## 2. The Clinical Stages of CKM Syndrome: A Progressive Continuum

The AHA’s classification of CKM syndrome is not merely a diagnostic checklist but a roadmap of progressive physiological decay. Staging the syndrome from 0 to 4 allows clinicians to pinpoint where a patient’s location on the spectrum of metabolic-to-cardiovascular collapse.

### 2.1. The Staging Framework

The progression of CKM syndrome is defined by the accumulation of risk factors and the eventual emergence of end-organ damage. Stage 0 includes individuals with healthy weight and normal glucose/blood pressure, where the focus here is entirely on primordial prevention. The shift to Stage 1 is defined by excess adiposity and metabolic priming; notably, even in the absence of overt diabetes, this stage reflects a state of “mitochondrial priming,” during which adipose tissue initiates the release of pro-inflammatory adipokines and free fatty acids [11]. As the syndrome advances to Stage 2, patients develop manifests clinical markers such as type 2 diabetes, hypertension, or hypertriglyceridemia, which impose chronic stress on the vascular endothelium and the renal filtration barrier. This pathological evolution culminates in Stage 3, representing a critical transition to subclinical organ failure, often evidenced by early-stage CKD or echocardiographic evidence of structural heart disease, such as left ventricular hypertrophy, despite the absence of overt symptoms. The final stage, Stage 4, involves overt disease, further stratified into Stage 4a (without kidney failure) and Stage 4b (with kidney failure or end-stage renal disease [ESKD]), where cardiovascular events—such as myocardial infarction or heart failure—become the primary drivers of morbidity (Figure 1).

### 2.2. Epidemiological Burden and the Aging Population

The prevalence of CKM syndrome has reached epidemic proportions, particularly in aging populations. Emerging evidence suggests that a staggering majority of adults in developed nations now fall somewhere along the CKM spectrum [12].

Epidemiological surveys indicate that approximately 90% of adults in the United States meet the criteria for at least stage 1 CKM syndrome [13], with the risk increasing exponentially alongside advancing age [14]. In populations over 65, the prevalence of stage 3 and stage 4 disease rises sharply, reflecting the cumulative effect of lifelong metabolic stress and the progressive depletion of mitochondrial reserve—a biological decline increasingly recognized as mitosenescence [15,16].

Recent studies also highlight a concerning shift in the “compression of morbidity” paradigm; while lifespans have extended, patients are enduring more prolonged periods of illness and multi-organ dysfunction [17]. In aging cohorts, the presence of Stage 3 CKD escalates the risk of cardiovascular mortality by two- to four-fold compared to individuals with preserved renal function [18]. These data underscores that CKM syndrome is not merely a cluster of co-morbidities, but rather a singular, integrated health crisis rooted in shared systemic failure.

The rising global burden of metabolic disease can also be interpreted through an evolutionary and bioenergetic framework centered on mitochondrial variation. Historical mitochondrial DNA (mtDNA) polymorphisms were shaped by the global dispersal of modern humans, where specific variants were positively selected to optimize energy production under specific environmental pressures, such as ambient temperature and nutrient availability. For instance, adaptation to colder climates favored mitochondrial variants that enhanced thermoregulation and tissue-specific bioenergetic capacity, often by modulating oxidative phosphorylation efficiency to favor heat generation. However, these same genomic features that once fine-tune energy production may now heighten the generation of deleterious metabolic byproducts, predisposing individuals to mitochondria to dysfunction and thereby influencing the onset and severity of adult metabolic diseases [19].

Since the Industrial Revolution, profound nutritional, epidemiological, and demographic transitions have created a mismatch between historically adaptive mitochondrial functions and the modern environment. Sustained caloric excess, physical inactivity, and the increased longevity subject mitochondria to chronic nutrient overload. This results in excessive electron transport chain flux, increased reactive oxygen species (ROS) production, and impaired metabolic flexibility. With aging, the progressive decline in mitochondrial quality control and accumulation of damage further amplifies these stresses, unmasking the pathological latent consequences of previously advantageous variants. Consistent with the theory of antagonistic pleiotropy, these evolutionary adaptations may now contribute to an increased susceptibility to non-communicable diseases, including cancer and coronary artery disease, with mounting evidence suggesting that common mtDNA haplogroups significantly influence disease pathogenesis and severity of cardio vasculars [20,21].

## 3. Mitochondria as the Engine of the CKM Vicious Cycle

The transition from metabolic homeostasis to CKM syndrome represents a fundamental collapse in energy governance. Although clinical manifestations typically emerge as hyperglycemia or hypertension, the foundational dysfunction originates within the mitochondria. This marks the onset of a “vicious cycle,” in which chronic nutrient excess drives progressive bioenergetic failure [22].

### 3.1. The Metabolic Component: Lipotoxicity and the Uncoupling Crisis

The early stages of CKM syndrome (Stages 1 and 2) are characterized by a nutrient-saturated environment that overwhelms cellular processing capacity. In the context of insulin resistance, the primary metabolic insult is lipotoxicity—the ectopic accumulation of non-esterified fatty acids (NEFAs) and toxic lipid derivatives, such as ceramides, in non-adipose tissues [23,24,25].

When mitochondria are inundated with an excess of lipid substrates, the β-oxidation pathway becomes saturated [26,27]. This over-nutrition forces mitochondrial membrane hyperpolarization, placing the electron transport chain (ETC) in a state of chronic incapacitate [28,29]. As the proton gradient across the inner membrane becomes excessively steep, electrons’ flow is impeded, leading to electron leakage from complexes I and III. These wayward electrons react prematurely with molecular oxygen to generate reactive oxygen species (ROS), primarily the superoxide radical [30,31].

To mitigate this mounting oxidative pressure, cells often upregulate uncoupling proteins (UCPs), dissipating the proton motive force as heat rather than harnessing it for ATP synthesis [32,33]. This lipid-driven pathological decoupling fundamentally limits the generation of high-energy phosphates, crippling the energy-intensive processes of ventricular relaxation and renal proximal tubule resorption [25].

**Figure 2:**
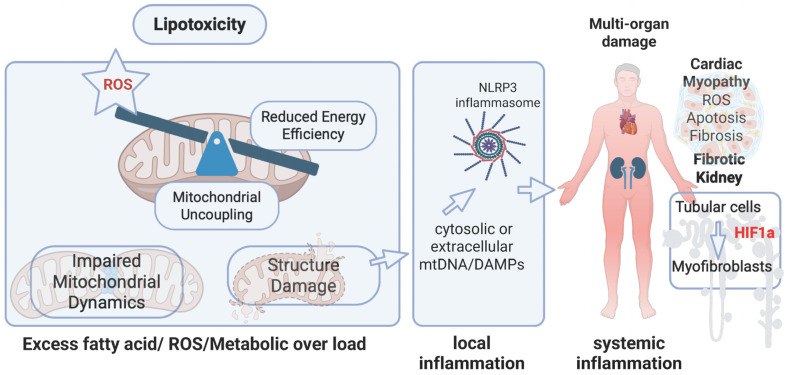
Mitochondrial Dysfunction as the Unifying Driver of Multi-Organ Damage in CKM Syndrome. Schematic representation of the proposed role of mitochondrial dysfunction in linking metabolic stress to systemic inflammation and organ injury in cardiovascular–kidney–metabolic (CKM) syndrome. Chronic nutrient excess and lipotoxicity increase fatty acid flux and reactive oxygen species (ROS) production, leading to mitochondrial bioenergetic imbalance characterized by reduced energy efficiency, mitochondrial uncoupling, impaired mitochondrial dynamics, and structural damage. These alterations promote further ROS generation and metabolic overload, establishing a self-amplifying cycle of mitochondrial dysfunction. Damaged mitochondria release mitochondrial DNA (mtDNA) and damage-associated molecular patterns (DAMPs) into the cytosol and extracellular space, activating inflammatory pathways, including the NLRP3 inflammasome, and initiating local inflammation. This response propagates into systemic inflammation, contributing to multi-organ injury. In the heart, these processes drive cardiomyocyte dysfunction, apoptosis, and fibrosis, while in the kidney, mitochondrial stress promotes tubular injury, HIF-1α activation, and myofibroblast transition, ultimately leading to fibrotic remodeling. Together, these interconnected pathways highlight mitochondria as a central integrative hub linking metabolic overload to inflammation and organ damage in CKM syndrome.

Crucially, the consequences of this mitochondrial lipotoxicity extend beyond classical oxidative stress to include ferroptosis—a form of regulated cell death driven by the toxic accumulation of lipid peroxides. Recent genome-wide association studies (GWAS) have identified convergent genetic signals in patients with CKM syndrome, specifically highlighting the mitochondrial beta-lactamase (LACTB) and phospholipase A2 group VI(PLA2G6) as core cardiometabolic and kidney risk genes. Within the mitochondria, LACTB cleaves and activates PLA2G6 to regulate oxidized phospholipid metabolism, thereby protecting renal and metabolic cells from ferroptosis induced by oxidized phosphatidylethanolamine (oxPE). When these structural and genetic mitochondrial safeguards fail, the resulting bioenergetic collapse and lipid-driven cell death trigger the systemic release of damage-associated molecular patterns (DAMPs), effectively priming the interconnected cardiorenal systems for terminal injury [34,35,36] (Figure 2).

### 3.2. The Cardiovascular Toll: From ATP Deficiency to Heart Failure

Within the CKM axis, the heart functions as the final common pathway of systemic failure. Unlike the kidney, which possesses a degree of metabolic flexibility, the myocardium is constrained by its relentless, high energy demand for ATP to support contractile activity. In CKM Stage 4, the heart is effectively caught between two pathological forces: widespread oxidative stress originating from systemic metabolic and renal disturbances, and intrinsic mitochondrial dysfunction within the cardiomyocytes themselves [37,38,39].

### 3.3. ATP Deficiency and Diastolic Dysfunction

The earliest sign of cardiac mitochondrial failure in CKM syndrome is often impaired myocardial relaxation (diastolic dysfunction), characteristic of heart failure with preserved ejection fraction (HFpEF) [40,41]. Active relaxation (the sequestration of calcium back into the sarcoplasmic reticulum via SERCA pump) is a highly ATP-dependent process [42]. When mitochondria fail to meet this energetic demand, cytosolic calcium levels remain elevated during diastole, causing myocardial stiffness and reduced compliance [43].

Oxidative stress and structural remodeling as mitochondrial ROS production peaks trigger the opening of the mitochondrial permeability transition pore (mPTP) [44]. This event is bioenergetically catastrophic; it leads to mitochondrial swelling, the release of cytochrome c into the cytosol, which ultimately orchestrates cardiomyocyte apoptosis [45,46]. The loss of functional muscle cells is followed by the deposition of collagen (fibrosis). This structural remodeling is the point of no return in CKM progression, leading to the dilated cardiomyopathy and the systolic failure seen in HFrEF [46].

### 3.4. The Renal Connection: Vulnerability of the Proximal Tubule

The kidney is among the most metabolically demanding organs in the human body. The proximal convoluted tubule (PCT), responsible for the bulk of solute reabsorption, relies almost exclusively on oxidative phosphorylation and possesses the second-highest mitochondrial density, trailing only the heart [47].

In the context of CKM syndrome, the kidney acts as a metabolic amplifier. Under the strain of chronic hyperglycemia and lipotoxicity, renal mitochondria undergo profound structural changes [48]. Dysfunctional mitochondria in the PCT do more than merely lose efficiency; they begin to release mtDNA into the cytosol [49]. Because mtDNA lacks protective histones and is rich in unmethylated CpG motifs, innate immune system perceives it as a danger signal—DAMP—which activates the NLRP3 inflammasome [50].

This activation triggers a cascade of both intra-renal and systemic inflammation. Chronic mitochondrial ROS production in the kidney stabilizes HIF-1 α, even in the absence of true hypoxia, leading to the transformation of tubular cells into myofibroblasts [51,52]. This metabolic-to-fibrotic transition is the hallmark of CKM Stage 3. As renal mitochondrial health declines, the kidney’s ability for sodium and water homeostasis is compromised, directly escalating the hemodynamic after load on the heart [53] (Figure 2).

### 3.5. Molecular Mechanisms of Failure: The Breakdown of Mitochondrial Quality Control

The transition from a healthy metabolic state to the multi-organ collapse of CKM syndrome is governed by a breakdown in mitochondrial homeostasis [54]. In healthy cells, mitochondria exist in a highly dynamic network, constantly reshaping themselves and undergoing quality control to ensure that only functional organelles remain [55]. In CKM syndrome, this equilibrium is shattered, leading to an accumulation of dysfunctional mitochondria.

### 3.6. Mitochondrial Dynamics: The DRP1/Mitofusin Imbalance

Mitochondrial morphology is governed by the opposing forces of fission (division) and fusion (joining). Under a healthy physiological state, fusion—mediated by Mitofusins 1 and 2 (MFN1/2) for the outer membrane and OPA1 for the inner membrane—facilitates the dilution of localized damage and the exchange of metabolic components [55].

In the CKM syndrome, chronic nutrient overload and oxidative stress shift the balance toward excessive fission [56]. This process is primarily driven by the excessive recruitment of dynamin-related protein 1 (DRP1) to the mitochondrial outer membrane [57]. This DRP1-mediated imbalance has two impacts on cardiac and renal dysfunction. First, excessive fission leads to mitochondrial fragmentation. These smaller, fragmented mitochondria exhibit diminished ATP production capacity and a heightened propensity for ROS leakage. Secondly, fragmented mitochondria in the CKM heart fail to maintain the spatial organization required for efficient excitation–contraction coupling [58]. Similarly, in the kidney, DRP1-mediated fragmentation of proximal tubule mitochondria serves as a precursor to tubular cell apoptosis and subsequent interstitial fibrosis [59].

### 3.7. Mitophagy: The Failure of Organelle Recycling

To maintain cellular health, damaged mitochondria are selectively eliminated via mitophagy—a specialized form of autophagy—to recycle the organelle. The major pathway for this process is the PINK1/Parkin axis [60,61,62].

Under normal conditions, PINK1 is rapidly degraded. However, when a mitochondrion loses its membrane potential due to CKM-related stress, stabilized PINK1 on the outer membrane recruits the E3 ligase Parkin. This marks the mitochondrion for autophagosomal degradation [63,64].

In CKM syndrome, this recycling pathway is frequently impaired, leading to the accumulation of deformity mitochondria that are bioenergetically and metabolically toxic [65,66]. These damaged organelles serve as a source of mitochondrial DNA (mtDNA) leakage into the cytoplasm. Because mtDNA contains unmethylated CpG motifs, it activates the cGAS-STING pathway and the NLRP3 inflammasome. This innate immune activation drives the systemic inflammation that characterizes Stage 3 and 4 CKM syndrome [67,68,69,70,71,72,73].

### 3.8. Sensing and Signaling: Mitochondrial AKT1 and Organ Cross-Talk

**Figure 3:**
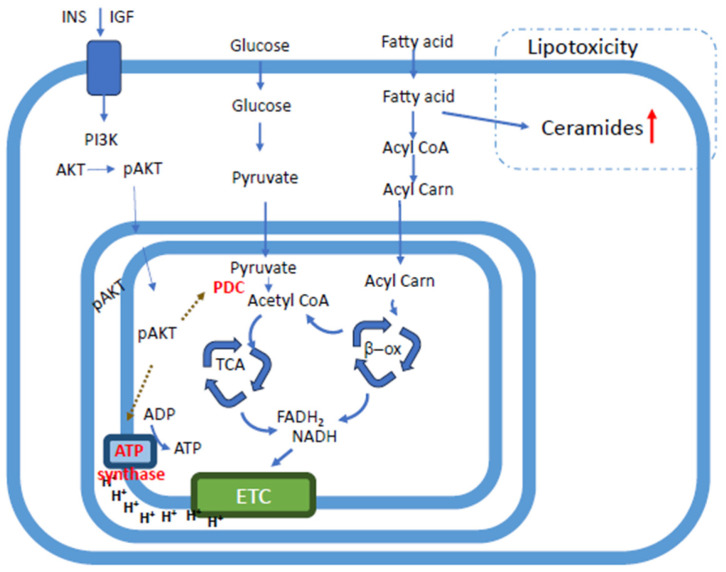
Integrated Mitochondrial AKT1 Signaling and Metabolic Flux in CKM Syndrome. Schematic illustration of mitochondrial substrate utilization and bioenergetic remodeling under conditions of metabolic overload in CKM syndrome, highlighting the central regulatory role of AKT signaling. In response to nutrient availability and insulin signaling, activation of AKT functions as a key upstream regulator of cellular metabolism, modulating glucose uptake, glycolytic flux, and mitochondrial substrate entry. Under physiological conditions, AKT signaling promotes coordinated substrate utilization, facilitating the conversion of pyruvate through the pyruvate dehydrogenase complex (PDC) and supporting efficient tricarboxylic acid (TCA) cycle activity. Reducing equivalents (NADH and FADH_2_) generated from these pathways drive the electron transport chain (ETC), enabling ATP production via oxidative phosphorylation. In the setting of chronic nutrient excess and insulin resistance, dysregulation of AKT signaling disrupts this balance. Impaired or maladaptive AKT activity alters PDC regulation and substrate preference, contributing to metabolic inflexibility, excessive substrate flux, and mitochondrial redox imbalance. These changes promote inefficient electron transfer within the ETC, increased reactive oxygen species production, and reduced ATP synthesis. Collectively, AKT-mediated signaling dysfunction links extracellular metabolic cues to mitochondrial bioenergetic failure, reinforcing a self-amplifying cycle of mitochondrial stress that contributes to CKM progression.

While the phosphatidylinositol-3-kinase (PI3K)/AKT pathways are well-known for its cytosolic functions, its translocation into the mitochondria is essential for regulating the ETC and maintaining organelle integrity [74,75,76]. Emerging evidence suggests that the mitochondrial translocation of AKT1 is an essential protective mechanism in both the heart and kidneys against CKM-related injury [37,48,77]. Under CKM-related metabolic stress, mitochondria in the renal tubules exhibit a predictable trajectory of failure by three distinct phases. First, ultrastructural degradation: mitochondria undergo morphological deformation, including simultaneous pathological elongation and fragmentation. There is a significant increase in aberrant mitochondria exhibiting matrix hyperlucency, loss of electron-dense matrix, and structural disruption of the outer membranes [48]. Second, cristae disorganization: a marked reduction in both cristae length and cristae density severely compromises the available surface area for energy production, leading to a collapse in bioenergetic capacity. Third, AKT1 signaling translocation: in response to metabolic stress, activated AKT1 (pAKT1) translocates into the mitochondria compartments of renal tubular cells. While this translocation initially serves as a protective mechanism for cell survival, its disruption exacerbates renal tubular injury and cumulates in programmed cell death (Figure 3).

Insulin-stimulated phospho-AKT1 (pAKT1) translocates to all mitochondrial sub-compartments—the inner and outer membranes and the intermembrane space—where it promotes efficient oxidative phosphorylation and ATP production. Under the metabolic stress typical of CKM syndrome, such as diabetes and insulin resistance, this translocation is severely impaired, precipitating the development of diabetic cardiomyopathy. The absence of mitochondrial AKT1 signaling triggers several outcomes. Cardiomyocytes exhibit a loss of organized mitochondrial cristae and reduce overall mitochondrial size [77]. Mitochondrial AKT1 is essential for the proper assembly and maintenance of the ATP synthase. Its absence leads to structural malformation of the ATP synthase, compromising both the energy-producing catalytic efficiency and its role in reinforcement of the cristae. Disruption of the mitochnondrial AKT1 translocation reduces oxidative phosphorylation efficiency and increases uncoupled respiration (proton leak), leading to a significant decline in myocardial ATP content. These cellular defects manifest as left ventricular hypertrophy and significant cardiac fibrosis. Conversely, the targeted activation of mitochondrial AKT1 has been shown to protect the heart and exert systemic metabolic benefits, including attenuating cardiac hypertrophy, preserving cristae structure, and improving cardiac contractile function in CKM syndrome. In cell respiration, ATP production is nearly doubled with lower oxygen consumption, suggesting a shift toward a more tightly coupled and efficient respiration. Enhancing this cardiac-specific mitochondrial AKT1 has pronounced 42% higher radiolabeled [18F] fluoro-4-thia-oleate (FTO) uptake and a 20% elevation in total energy expenditure.

While lipotoxicity and subsequent ferroptosis drive severe structural decay, aberrant amino acid metabolism also plays a critical, intersecting pathogenic role. Recent metabolomic profiling of patients with concomitant metabolic syndrome and heart failure has identified a pronounced defect in mitochondrial branched-chain amino acid (BCAA) catabolism, leading to systemic BCAA accumulation.

The initial steps of BCAA catabolism occur exclusively within the mitochondria, this defect is both a primary symptom and an exacerbator of mitochondrial insolvency. High levels of circulating BCAAs are known to chronically hyperactivate the mammalian target of rapamycin (mTOR) pathway. This mTOR hyperactivation subsequently induces profound insulin resistance and suppresses protective PI3K-AKT signaling through established negative feedback loops. Therefore, the protective translocation of pAKT1 to the mitochondria does not operate in a vacuum; it actively competes against a hostile, mTOR-activated environment driven by both lipid and amino acid overload.

The clinical relevance of addressing these broader mitochondrial metabolic networks is rapidly emerging. A 2025 study demonstrated that pharmacologically restoring BCAA catabolism—specifically via the inhibition of branched-chain ketoacid dehydrogenase kinase (BDK)—significantly improves kidney function and attenuates renal decline in preclinical models of CKM syndrome [78].

## 4. Current Pharmacological Interventions: Restoring Mitochondrial Economy

The clinical management of CKM syndrome has been revolutionized by three cornerstone pharmacological classes: Sodium–glucose cotransporter 2 inhibitors (SGLT2i), GLP-1 receptor agonists (GLP-1RAs)/dual glucose-dependent insulinotropic polypeptides (GIP)/GLP-1RA, and non-steroidal mineralocorticoid receptor antagonist (ns-MRAs). While these agents were originally designed for glycemic or proteinuria control, their most profound impacts—cardioprotection and renoprotection—are increasingly attributed to their ability to stabilize mitochondrial health (Table 1).

### 4.1. SGLT2 Inhibitors: Enhancing Bioenergetic Efficiency

SGLT2i (e.g., canagliflozin, empagliflozin, and dapagliflozin) originally developed as antidiabetic agents, have transcended their initial indications to become agents for cardiovascular and renal protection.

### 4.2. Shift in Substrate Utilization (The “Thrifty Substrate” Hypothesis)

In the heart and kidney of the CKM patients, mitochondria are often over-reliant on fatty acid oxidation, an oxygen-expensive process that generates high levels of ROS. SGLT2i induces a state of mild systemic ketosis. These ketone bodies, specifically β-hydroxybutyrate, provide a more bioenergetically efficient fuel source than fatty acids. This metabolic shift increases the ATP/oxygen ratio, effectively unloading the metabolic stress from struggling cardiomyocyte and renal tubular cells [102].

### 4.3. Mitigation of Oxidative Stress and Calcium Overload

Recent research has demonstrated that SGLT2i directly modulates the cardiac sodium–hydrogen exchanger (NHE) in the heart [103]. By attenuating intracellular sodium, these agents prevent mitochondrial calcium overload—a primary trigger for the opening of the mitochondrial permeability transition pore (mPTP) and subsequent programmed cell death [104]. Furthermore, SGLT2i have been shown to stimulate mitochondrial biogenesis via the PGC-1α pathway, essentially helping the cell healthy renewal [105].

### 4.4. GLP-1 Receptor Agonists: Protecting Structural Integrity

GLP-1RA (e.g., semaglutide, liraglutide, and dulaglutide) and GIP/GLP-RA (tirzeptide) address the complexities of CKM syndrome through a multi-faceted therapeutic approach. These agents effectively target upstream metabolic drivers by reducing adiposity, while simultaneously exerting direct, anti-inflammatory effects on the heart and kidneys.

### 4.5. Reducing Systemic and Local Inflammation

MtDNA leakage has been identified as a major driver of the inflammation characterizing CKM Stages 3 and 4 [57,67,106,107]. GLP-1 RAs effectively counter this progression by suppressing a broad spectrum of inflammation-related cytokines, including, ICAM-1, VCAM-1, IL-6, TNFα and IL-12 [108]. By stabilizing the mitochondrial membrane, these agents prevent the efflux of mitochondrial DAMPs (Damage-Associated Molecular Patterns) into the cytosol, thereby halting the pathological crosstalk between the kidney and the heart [109].

### 4.6. Preserving Mitochondrial Morphology

Emerging evidence suggests that GLP-1 signaling actively promotes mitophagy flux. By upregulating the PINK1/Parkin pathway, GLP-1 RAs facilitate the efficient clearance of damaged, ROS-leaking mitochondria before they can initiate pro-apoptotic signaling cascades [110]. This preservation of mitochondrial “quality control” may protect the high energy-demanding tissues, such as cardiomyocytes and renal proximal tubular cells, protecting them from the pathological transition toward fibrosis [111]. Furthermore, in the cardiovascular system, GLP-1 RAs have been shown to reduce epicardial fat volume—a significant source of local mitochondrial toxins that contribute to atrial fibrillation and heart failure [112].

### 4.7. Mineralocorticoid Receptor Antagonists: Arresting Fibro-Inflammation

While SGLT2i and GLP-1 RAs primarily address the metabolic and bioenergetic derangements of CKM syndrome, MRAs target the neurohormonal overactivation that drives structural decay. The mineralocorticoid receptor (MR) is not limited to the renal tubules; it is extensively expressed in cardiomyocytes, fibroblasts, and immune cells.

### 4.8. Non-Steroidal MRAs

The recent development of ns-MRAs, such as finerenone, represents a significant leap over traditional steroidal agents like spironolactone and eplerenone. In contrast to its predecessors, finerenone exhibits higher receptor selectivity and a more balanced distribution between cardiac and renal tissues. This pharmacological profile significantly mitigates the risk of hyperkalemia while maximizing organ-protective efficiency. Landmark clinical trials like FIDELIO-DKD and FINEARTS-HF have provided robust evidence that ns-MRAs substantially reduce the risk of cardiovascular death and heart failure hospitalizations across the CKM spectrum [89,113].

### 4.9. Mitochondrial Protection via MR Blockade

The mechanism by which MRAs preserve mitochondrial integrity is multifaceted, primarily centered on the inhibition of the oxidative burst. MR activation stimulates NADPH oxidase, a major source of cytosolic ROS [114]. This cytosolic ROS triggers a deleterious phenomenon known as “ROS-induced ROS release,” where mitochondrial membranes are depolarized, leading to a secondary burst of mitochondrial oxidative stress [115]. By antagonizing the MR, finerenone halts this pathological chain reaction, thereby preserving the mitochondrial membrane potential. Furthermore, the MRAs mitigate mitochondrial-to-fibrotic signaling. Chronic MR activation in the cardio-renal axis promotes the stabilization of the NLRP3 inflammasome [116].

## 5. Emerging Mitochondrial-Targeted Therapies: The Next Frontier

Despite the success of systemic metabolic therapies, a therapeutic gap persists for patients with advanced CKM syndrome whose mitochondrial damage has become autonomous and self-sustaining. The next generation of CKM medicine may focus on mitochondria-targeted-molecular agents specifically designed to penetrate the double-membrane barrier of the mitochondrion to exert direct effects on the ETC and the mitochondrial genome.

### Mitochondrial-Targeted Antioxidants: Precision Redox Control

Traditional antioxidants (e.g., Vitamin E or C) have largely failed to demonstrate clinical efficacy in clinical trials for CKM because they cannot achieve sufficient concentrations at the intramitochondrial sites where ROS are primarily generated [117]. Emerging therapies solve this pharmacological barrier via molecular engineering.

- MitoQ (mitoquinone): This compound utilizes a lipophilic triphenylphosphonium (TPP) cation to harness the mitochondrial membrane potential. Given that the mitochondrial matrix is negatively charged, MitoQ accumulates several hundred-fold within the organelle. By positioning a ubiquinone directly at the inner membrane, MitoQ effectively intercepts as electrons leakage from Complexes I and III, preventing the lipid peroxidation that drives the transition to CKM Stage 3 and subsequent renal fibrosis [118,119].
- Elamipretide (SS-31): Distinct from conventional radical scavengers, elamipretide is a tetrapeptide that binds specifically to cardiolipin, a signature phospholipid of the inner mitochondrial membrane. Cardiolipin is essential for the structural integrity of cristae and the formation of ETC complexes In CKM models, elamipretide prevents the oxidative modification of cardiolipin, thereby stabilizing the mitochondrial membrane potential and reducing the proton leak associated with the development of HFpEF [120,121].
- NAD+ Boosters: As CKM syndrome progresses, cellular levels of nicotinamide adenine dinucleotide (NAD+) undergo a precipitous decline—a state of NAD+ deficiency syndrome. This is catastrophic for the CKM axis because NAD+ is a mandatory co-factor for the sirtuin family of enzymes [122].

## 6. Limitations

Several limitations warrant consideration. First, direct clinical evidence linking mitochondrial dysfunction to cardiovascular–kidney–metabolic (CKM) syndrome as an integrated entity remains limited, reflecting the recent recognition of CKM as a unified clinical construct and the historical compartmentalization of cardiovascular, renal, and metabolic research. As a result, much of the available evidence derives from preclinical studies or clinical investigations focused on individual disease domains. Second, rigorous, scalable assessment of mitochondrial function in humans remains challenging, and standardized, clinically validated mitochondrial biomarkers are not yet widely established. Third, although accumulating data indicate that cornerstone therapies—including sodium–glucose co-transporter 2 inhibitors, glucagon-like peptide-1 receptor agonists, and non-steroidal mineralocorticoid receptor antagonists—exert important effects on mitochondrial biology, these mechanisms have not been prospectively evaluated as primary endpoints in large-scale clinical trials.

Notwithstanding these limitations, the consistent convergence of mechanistic and clinical evidence across organ systems supports a central role for mitochondrial dysfunction in CKM pathophysiology. This framework provides a biologically coherent basis for integrating multi-organ disease progression and therapeutic response. Future efforts should prioritize the development of validated mitochondrial biomarkers, incorporation of bioenergetic phenotyping into CKM staging, and the design of prospective trials that directly interrogate mitochondrial pathways. Advancing such a mitocentric approach may enable earlier intervention, refine risk stratification, and establish mechanism-based precision therapies for CKM syndrome.

## 7. Conclusions: The Mitocentric Future of CKM Care

In conclusion, the rising burden of metabolic diseases in aging populations can be understood as the consequence of an evolutionary mismatch in which mitochondria—once optimized for fluctuating environments and limited nutrient availability—are now chronically challenged by sustained caloric excess and reduced energy demand. This persistent bioenergetic overload promotes electron transport chain stress, excessive reactive oxygen species production, and progressive mitochondrial dysfunction, processes that are further exacerbated with age and contribute to the development of kidney and cardiovascular diseases. Notably, a substantial body of prior work has independently documented these mitochondrial abnormalities across cardiovascular, renal, and metabolic disorders; however, these findings have largely been interpreted within organ-specific contexts.

The formalization of CKM syndrome represents a significant shift in modern clinical medicine. By moving away from the treatment of isolated symptoms toward a more integrated systemic framework, the medical community increasingly recognizes the biological interconnectedness of multiple organ systems. As discussed throughout this review, the mitochondrion emerges as a central hub within this axis—not merely as a provider of ATP, but as a dynamic regulator of metabolic signaling that links nutrient excess, inflammatory responses, and progressive organ dysfunction. By synthesizing evidence across disciplines, this review extends existing models and positions mitochondrial dysfunction as a unifying mechanistic axis underlying CKM syndrome.

Importantly, this framework also highlights actionable opportunities for intervention. Lifestyle strategies that restore elements of ancestral energetic balance—such as caloric moderation, improved diet quality, regular physical activity, and maintenance of circadian alignment—can reduce mitochondrial substrate overload and enhance metabolic flexibility. These interventions may alleviate redox stress, improve mitochondrial efficiency, and preserve organ function, thereby lowering the risk of kidney dysfunction and heart failure. Framing disease prevention through the lens of mitochondrial bioenergetics not only provides mechanistic insight but also aligns with a growing body of clinical and translational evidence supporting metabolic and lifestyle interventions in CKM-related conditions. Collectively, this integrative framework provides a mechanistic basis for transitioning toward mitochondria-informed precision medicine in CKM syndrome.

## Figures and Tables

**Table 1:** Comparative Mitochondrial Effects of CKM Therapies. This table summarizes the molecular reprogramming and organ-protective mechanisms of the therapies for CKM syndrome.

	Sodium–Glucose Cotransporter-2 Inhibitors SGLT2i	Glucagon-Like Peptide-1 Receptor Agonists GLP-1 RAs	Non-Steroidal Mineralocorticoid Receptor Antagonist nsMRAs
Drug name	Empagliflozin [79,80,81,82] Dapagliflozin [83,84]	Semaglutide [85,86,87,88]	Finerenone [89]
	Metabolic Reprogramming of Mitochondria	Enhance Mitochondrial quality control	Suppress mitochondrial fibrosis signals
Key Mitochondrial Effects	Shift substrate use toward ketone bodies (β-hydroxybutyrate) and fatty acids that yield more ATP per oxygen [83,84] Reduce mitochondrial ROS generation [90] Improve mitophagy [91] Improve coupling efficiency of oxidative phosphorylation [92] Increase mitochondrial biogenesis signals [93]	Reduce metabolic stress [94] Improve insulin signaling to mitochondria [95] Reduce lipotoxic mitochondrial injury [96] Lower inflammatory ROS production [97]	Lowers the production of ROS in mitochondria [98] Enhances mitochondrial metabolism [99] Decreasing mitochondrial fragmentation and promoting healthy, functional mitochondria [100]
Target Organs	Restores mitochondrial function systemically, especially in heart and kidney [79,80,81,82,83,84]	These drugs influence various physiological systems, including the endocrine, cardiovascular, nervous, and gastrointestinal systems [85,86,87,88,101]	Primarily protect the kidneys and the cardiovascular system (heart and blood vessels) [89]

## Data Availability

No new data were created or analyzed in this study. Data sharing is not applicable to this article.
